# Gut microbiota in asthma: mechanisms, clinical evidence, and therapeutic opportunities

**DOI:** 10.3389/fcimb.2026.1842331

**Published:** 2026-07-23

**Authors:** Zhenan Ruan, Fang Sheng, Mali Lin, Shamin Wu, Chuanze Hu, Zhuoqing Shao, Honghua Hu, Lidan Xu

**Affiliations:** 1 Department of Pediatrics, Jinhua Maternal and Child Health Care Hospital, Jinhua, Zhejiang, China; 2 Department of Pediatrics, Jinhua Municipal Central Hospital, Jinhua, Zhejiang, China; 3 College of Pharmaceutical Sciences, Zhejiang University, Hangzhou, China; 4 Macquarie Medical School, Macquarie University, Sydney, NSW, Australia

**Keywords:** asthma, fecal microbiota transplantation, gut-lung axis, immunomodulation, intestinal microbiota, short-chain fatty acids

## Abstract

Asthma is a heterogeneous chronic airway inflammatory disease associated with high global prevalence and substantial clinical burden. Conventional therapies remain limited in controlling refractory phenotypes and preventing disease progression. The gut-lung axis has emerged as a fundamental regulatory network connecting intestinal homeostasis with pulmonary immune function, and mounting evidence has established a close mechanistic link between gut dysbiosis and the onset, persistence, and exacerbation of asthma. This review comprehensively integrates evidence from epidemiological investigations, animal models, clinical observational studies, and randomized controlled trials published between 2011 and 2025 to elucidate the crosstalk mechanisms of the gut-lung axis in asthma, characterize compositional and functional alterations of the gut microbiome, evaluate microbiota-targeted interventions such as probiotics, prebiotics, synbiotics, postbiotics, and fecal microbiota transplantation, and discuss current translational challenges. We highlight that the gut microbiota orchestrates airway inflammatory responses through fine-tuning immune cell differentiation, mediating microbial metabolite signaling, and maintaining intestinal barrier function, with discernible microbial signatures evident across allergic versus non-allergic and pediatric versus adult asthma phenotypes. Despite promising preclinical and preliminary clinical findings, causal evidence remains insufficient, and intervention heterogeneity limits clinical application. This review underscores the potential of microbiome-based precision strategies and identifies key directions for future mechanistic research and clinical translation.

## 1 Introduction

Asthma is one of the most prevalent chronic respiratory diseases worldwide, imposing a heavy and escalating global health burden despite continuous advances in therapeutic research (Masoli et al., 2004; Jayasooriya et al., 2025). Epidemiological data show that approximately 339 million people globally suffer from asthma, with an annual mortality of 436,000, highlighting a persistent gap between therapeutic progress and clinical outcomes (Jayasooriya et al., 2025).

Asthma is no longer regarded as a single clinical entity; decades of research have revealed its remarkable phenotypic, endotypic and clinical heterogeneity, which can be categorized by multiple classification criteria: clinically divided into pediatric and adult asthma; immunologically classified into Th2-high allergic asthma and non-Th2 asthma; and comorbidity-stratified into obesity-associated asthma, severe refractory asthma and steroid-resistant asthma. Each subtype possesses unique immunological landscapes, inflammatory cell infiltration patterns, clinical progression trajectories and treatment responses (Xie et al., 2024). Notably, the interplay between gut microbiota and asthma varies fundamentally across these heterogeneous subtypes: the dominant microbial taxa, core metabolites and immune regulatory axes differ greatly between allergic and non-allergic asthma, while pediatric and adult asthma show age-dependent microbial dysbiosis characteristics. Obesity-associated and severe asthma display the most severe gut microbial disturbance and metabolite depletion, which is closely linked to their typical steroid resistance and recurrent exacerbations (Heijink et al., 2014; Yerevanian and Soukas, 2019; Berkinbayeva et al., 2024; Xie et al., 2024).

This heterogeneity leads to common clinical dilemmas: a considerable proportion of patients present with glucocorticoid resistance, recurrent exacerbations despite standardized treatment, and progressive irreversible airway remodeling, including airway wall thickening, smooth muscle hyperplasia and hypertrophy, disordered collagen deposition in the reticular basement membrane, and goblet cell metaplasia with excessive mucus secretion (Heijink et al., 2014; Khalfaoui and Pabelick, 2023; Berkinbayeva et al., 2024; Gyawali et al., 2025). Once established, these structural alterations lead to fixed airflow limitations and accelerated decline in lung function. Current mainstream therapies, dominated by inhaled corticosteroids and long-acting bronchodilators, fail to reverse the natural course of the disease for most symptomatic, exacerbation-prone, and functionally impaired patients (Papi et al., 2022; LaForce et al., 2025; Rayner et al., 2025). These clinical dilemmas underscore a fundamental knowledge gap: we lack a mechanistic understanding of how distinct asthma endotypes differentially engage the gut-lung axis, which limits our ability to develop subtype-specific interventions.

The gut-lung axis, an intricate bidirectional communication network between the intestine and lungs, has emerged as a novel paradigm for addressing this knowledge gap (Özçam and Lynch, 2024; Burrows et al., 2025). A growing body of evidence confirms that perturbations in the gut microbiota are closely associated with the susceptibility, progression, and treatment response of respiratory diseases. For asthma specifically, epidemiological studies have demonstrated that early-life risk factors significantly increase the risk of childhood asthma, including cesarean section delivery, antibiotic exposure during infancy, lack of breastfeeding, environmental tobacco smoke exposure, and reduced microbial diversity during the first 100 days of life, indicating that microbial colonization during critical developmental windows programs long-term immune maturation (Boulund et al., 2025).

Microbial metabolites are the core mediators of the gut-lung axis. Short-chain fatty acids (SCFAs) produced by dietary fiber fermentation and tryptophan-derived indole compounds enter the systemic circulation, regulate lung-resident immune cell function, promote regulatory T cell (Treg) differentiation, inhibit inflammatory cascades, and enhance epithelial barrier integrity (Hu et al., 2024; Li et al., 2024; Rowland et al., 2025; Yu et al., 2025). However, several critical controversies and knowledge gaps remain in this rapidly evolving field. First, the causal direction remains unresolved: whether gut dysbiosis is a primary driver of asthma pathogenesis or a secondary epiphenomenon of established disease. Second, mechanistic details are incomplete: the precise molecular mechanisms of metabolite-receptor interactions, dose-dependent effects, and spatiotemporal dynamics of gut-to-lung signaling remain poorly characterized. Third, translational limitations persist: most clinical studies are observational rather than mechanistic, and animal models cannot fully recapitulate the complexity of human microbial communities and environmental interactions (Özçam and Lynch, 2024; Burrows et al., 2025).

**Figure 1:**
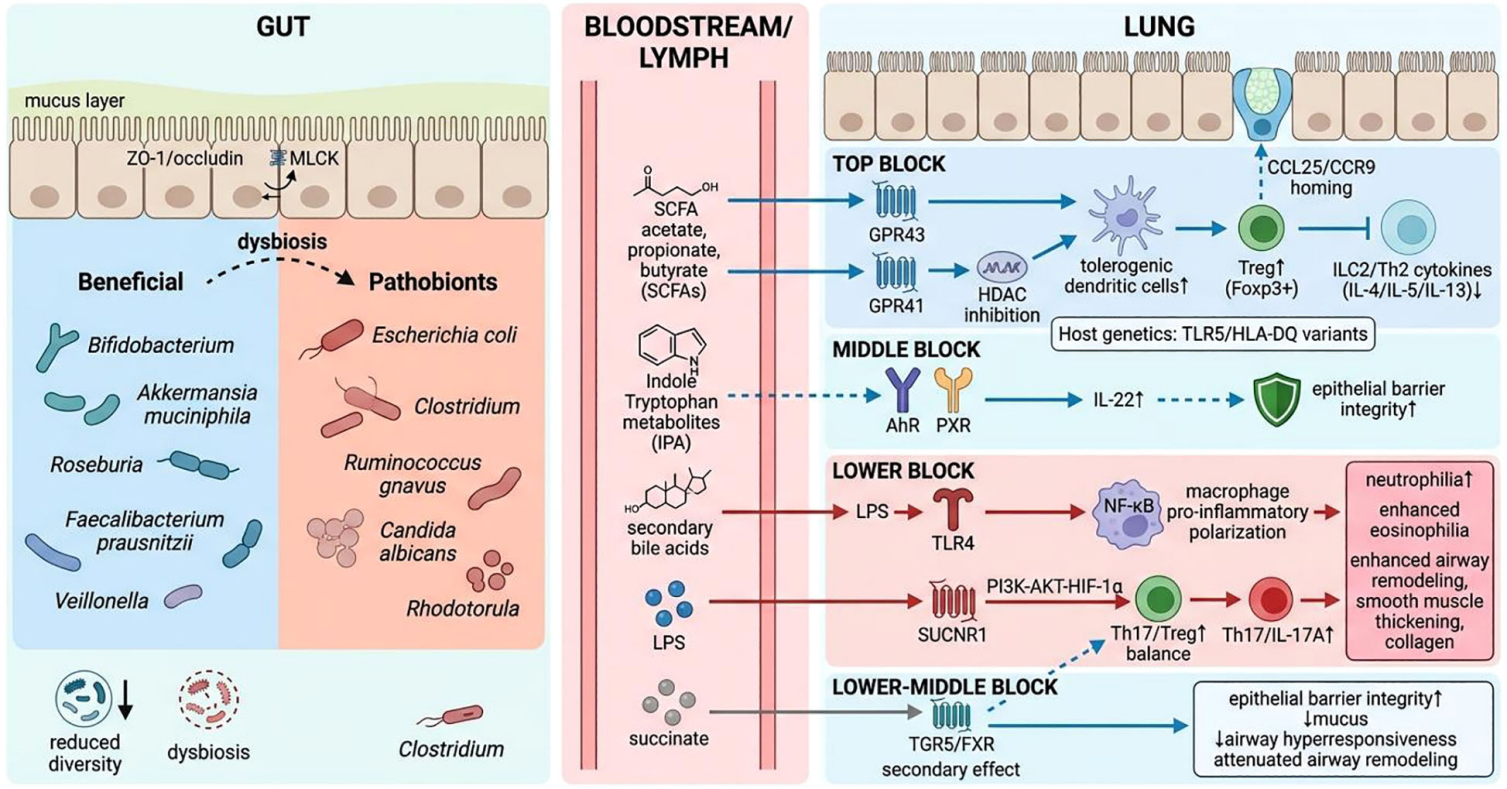
The gut-lung axis in asthma: intestinal dysbiosis, pulmonary immune modulation, and therapeutic opportunities.

This review comprehensively integrates the relationship between gut microbiota and asthma pathogenesis, synthesizing evidence from clinical epidemiology, immunological mechanisms, and emerging therapeutic strategies (see **Figure 1**). Unlike previous reviews that focus primarily on associative evidence, we emphasize mechanistic stratification across asthma endotypes and critically evaluate the translational gap between preclinical findings and clinical heterogeneity. We first elaborate on the developmental origins of gut microbial dysbiosis and its predictive value for asthma susceptibility across the lifespan. We then dissect the molecular mechanisms of microbe-host communication, focusing on metabolite-sensing pathways that regulate systemic immune homeostasis. Furthermore, we critically evaluate the translational potential of microbiota-based interventions, including probiotics, prebiotics, postbiotics, and fecal microbiota transplantation (FMT). Finally, we summarize the current methodological challenges and propose future directions to advance from associative observations to mechanistic validation and precision therapy. By integrating the latest evidence and acknowledging existing limitations, this review aims to provide a rigorous theoretical foundation for microbiome-targeted therapeutic innovation in asthma management.

## 2 Pathological mechanisms of asthma

**Figure 2:**
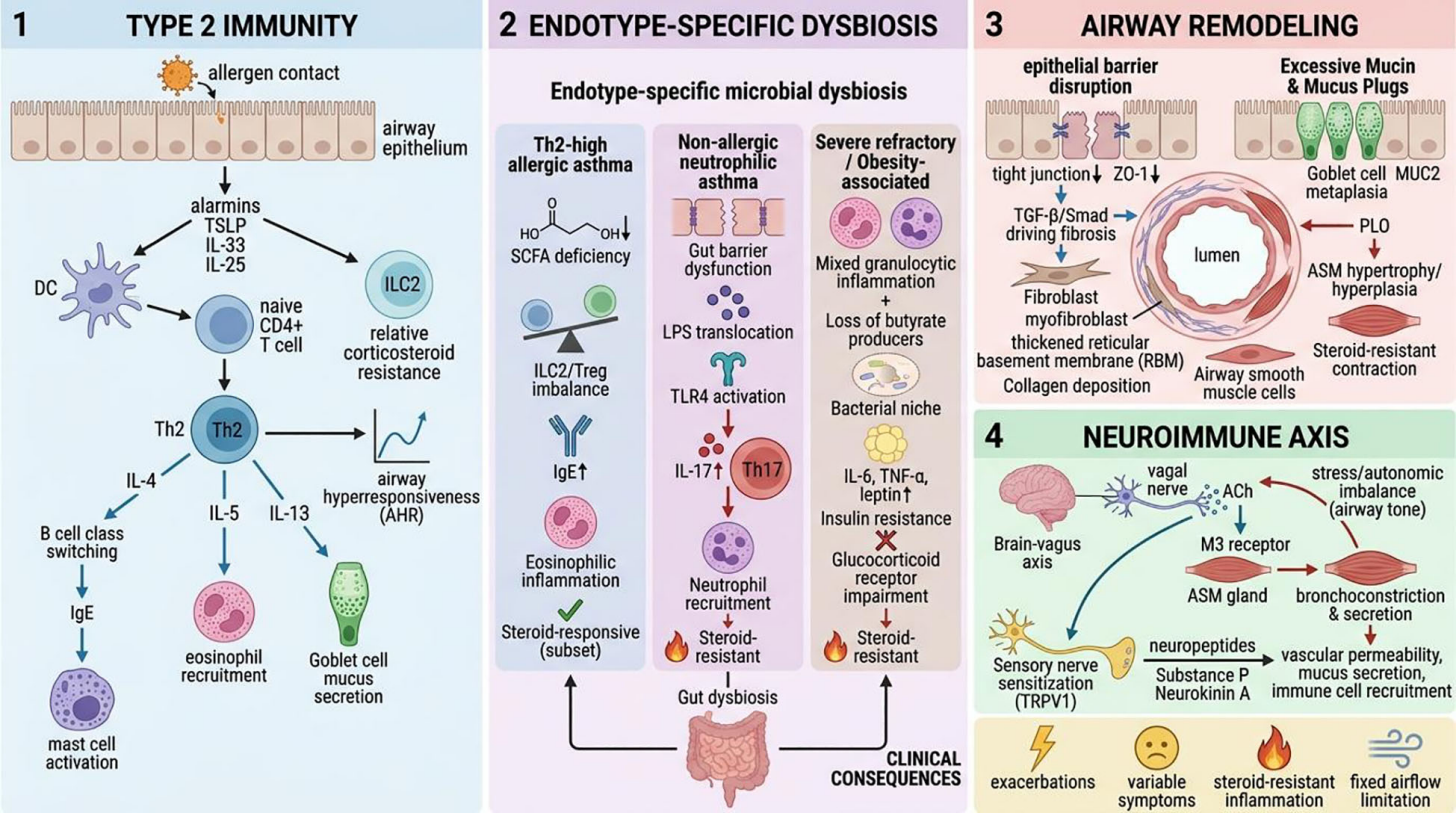
Core pathological mechanisms of asthma.

Elucidating how intestinal microbiota modulates the fundamental pathophysiological mechanisms of asthma (**Figure 2**), encompassing immune-inflammatory cascades, airway structural alterations, and neuroimmune interactions, provides essential context for understanding the gut-lung axis as a therapeutic target (Ogulur et al., 2025).

### 2.1 Microbial regulation of type 2 immunity

The T helper 2 (Th2) immune response serves as the core driver of allergic asthma pathogenesis. Upon allergen exposure, airway epithelial cells release alarmins including thymic stromal lymphopoietin (TSLP), interleukin-33 (IL-33), and interleukin-25 (IL-25), which activate dendritic cells (DCs) to induce naive CD4^+^ T cell differentiation into effector Th2 cells (Schmitt et al., 2024). Activated Th2 cells trigger a type 2 cytokine cascade dominated by interleukin-4 (IL-4), interleukin-5 (IL-5), and interleukin-13 (IL-13). IL-4 and IL-13 drive B cell immunoglobulin E (IgE) class switching, while IL-5 recruits and activates eosinophils, leading to airway hyperresponsiveness and tissue remodeling (Castillo et al., 2018; Domingo et al., 2025; Sahnoon et al., 2025). Type 2 innate lymphoid cells (ILC2) provide an alternative pathogenic pathway independent of adaptive immunity, secreting cytokines rapidly upon initial exposure and exhibiting corticosteroid resistance that maintains persistent inflammation in severe asthma (Ogulur et al., 2025).

Intestinal microbiota critically modulates these type 2 pathways through metabolite-mediated mechanisms. SCFA deficiency, particularly butyrate depletion resulting from reduced *Faecalibacterium prausnitzii* (*F. prausnitzii*) and Roseburia abundance, compromises intestinal barrier integrity and permits increased lipopolysaccharide (LPS) translocation, which synergizes with Th2 cytokines to amplify eosinophilic airway inflammation (Miquel et al., 2013). Butyrate directly suppresses ILC2 proliferation and GATA3 expression through histone deacetylase (HDAC) inhibition, attenuating type 2 cytokine production (Thio et al., 2018). Concurrently, SCFAs promote IL-10-producing Tregs and regulatory B cells that suppress mast cell degranulation and IgE production, thereby establishing a critical checkpoint in the gut-lung immunoregulatory axis (Verma et al., 2024).

### 2.2 Endotype-specific microbial dysbiosis

Beyond the modulation of shared type 2 pathways, the immunopathological mechanisms driven by gut microbiota differ distinctly between core asthma endotypes. Classic Th2-high allergic asthma is predominantly modulated by SCFA deficiency and subsequent ILC2/Treg imbalance, which promotes IgE production and eosinophilic inflammation (Kabil et al., 2024). By contrast, non-allergic neutrophilic asthma is mainly triggered by gut barrier dysfunction and LPS translocation: the expansion of Gram-negative bacteria elevates circulating LPS, activates TLR4 signaling, and induces excessive IL-17 secretion and neutrophil recruitment, forming a Th17-dominant inflammatory microenvironment (McAlees et al., 2015). Severe refractory asthma often presents as mixed granulocytic inflammation, combining the above two pathways, accompanied by the most significant loss of beneficial butyrate-producing bacteria (Druszczynska et al., 2024). Obesity-associated asthma represents a particularly refractory endotype wherein adipose tissue-driven low-grade inflammation synergizes with microbial dysbiosis. elevated circulating IL-6, TNF-α, and leptin amplify both eosinophilic and neutrophilic airway inflammation, while obesity-induced insulin resistance impairs glucocorticoid receptor signaling, contributing to steroid resistance (Moore et al., 2010; Vernooy et al., 2010; Liu et al., 2017).

### 2.3 Microbial modulation of airway remodeling

Airway structural alterations constitute the pathological substrate for chronicity and irreversible airflow limitation, and emerging evidence indicates that intestinal microbiota-derived signals participate in these processes. Environmental stimuli downregulate tight junction proteins including occludin and zonula occludens-1 (ZO-1), weakening epithelial barrier integrity and facilitating continuous allergen entry (Heijink et al., 2020). SCFAs, particularly butyrate, enhance epithelial tight junction expression and promote mucus barrier integrity through MUC2 upregulation, thereby attenuating allergen penetration and subsequent inflammatory cascades (Burger-van Paassen et al., 2009). Inflammatory cascades drive goblet cell hyperplasia and metaplasia, promoting excessive mucin production and pathological mucus plug formation (Boonpiyathad et al., 2019). Airway smooth muscle cells undergo phenotypic transformation toward a synthetic and proliferative state, enhancing contractile responsiveness while diminishing sensitivity to relaxants (Camoretti-Mercado and Lockey, 2021). This structural transformation directly mediates glucocorticoid-resistant contraction in severe asthma (Ramos-Ramírez and Tliba, 2022). Microbial metabolites may modulate smooth muscle reactivity through GPR41/GPR43-dependent signaling, though this pathway remains incompletely characterized in human asthma (Tan et al., 2017). Subepithelial fibrosis progresses through fibroblast activation and myofibroblast differentiation, driven by TGF-β/Smad signaling and resulting in collagen deposition and airway wall thickening (Li et al., 2010; Wen et al., 2024; Yang et al., 2025). Butyrate has been shown to inhibit TGF-β-induced fibroblast activation and collagen synthesis *in vitro*, suggesting a potential protective role against airway fibrosis that warrants validation in clinical settings (Ouyang et al., 2025).

### 2.4 Gut-brain-lung neuroimmune axis

Neuroimmune interactions constitute an important regulatory layer shaping asthma exacerbations, and the intestinal microbiota exerts profound influence on these pathways through multiple mechanisms. Cholinergic hyperactivity increases vagal efferent activity, augmenting acetylcholine release and activating M3 muscarinic receptors on airway smooth muscle to mediate bronchoconstriction (Maclagan and Barnes, 1989; Datsi et al., 2021; Agache et al., 2025). The gut microbiota modulates vagal tone through the production of neuroactive metabolites including γ-aminobutyric acid (GABA) and serotonin, as well as SCFA-mediated activation of enteroendocrine cells, establishing a microbial-gastric-vagal reflex that may influence airway cholinergic tone (Bravo et al., 2011). Sensory nerve sensitization through TRPV1 amplification lowers the threshold for irritant responsiveness, promoting neuropeptide release that facilitates bronchoconstriction and type 2-biased inflammation (Kistemaker et al., 2014; Devos et al., 2016; Kim et al., 2024). Bacterial products including LPS and peptidoglycan can directly sensitize TRPV1-expressing nociceptors, suggesting a mechanism by which intestinal microbial translocation may amplify airway sensory nerve hyperreactivity (Peng et al., 2009). Neurogenic inflammation through axonal reflexes induces peripheral tachykinin release, increasing vascular permeability and inflammatory cell recruitment (Li et al., 2019). The brain-to-airway axis links stress and emotion to asthma control through hypothalamic-pituitary-adrenal and autonomic pathways, with autonomic imbalance favoring vagal predominance and cholinergic bronchoconstriction during emotional arousal (Nassenstein et al., 2008; Rapp et al., 2023). The gut microbiota critically shapes stress responsivity and HPA axis function through the production of corticotropin-releasing hormone analogs and modulation of circulating cortisol levels, implicating intestinal dysbiosis as a potential amplifier of stress-induced asthma exacerbations (Sudo et al., 2004).

In summary, the heterogeneous immunological, structural, and neuroimmune pathological characteristics of different asthma subtypes are closely coupled with distinct gut microbial dysbiosis patterns. The gut-lung axis mediates divergent pathological cascades across asthma phenotypes, which lays a theoretical foundation for exploring subtype-specific microbial markers and targeted therapies.

These pathological mechanisms, including immune-inflammatory cascades, airway remodeling, and neuroimmune dysregulation, collectively create a therapeutic ceiling for conventional pharmacotherapy. The gut-lung axis offers a fundamentally distinct entry point: by modulating systemic immune priming, metabolite availability, and neuroimmune tone, intestinal microbiota interventions may address upstream drivers of airway pathology that are refractory to inhaled therapies. This mechanistic rationale underscores the strategic value of microbiota-based approaches as complementary or alternative strategies for asthma management.

## 3 Gut microbiota and metabolites in asthma: from composition to function

The gut-lung axis mediates the pathological mechanisms described in Section 2 through specific microbial taxa and their bioactive metabolites. This section delineates the compositional alterations of intestinal microbiota in asthma and the functional mechanisms by which microbial products regulate pulmonary immunity, establishing the microbial foundation for subsequent therapeutic discussions.

### 3.1 Dysbiotic features in asthma

Clinical metagenomic studies have identified consistent patterns of intestinal dysbiosis in asthmatic patients across age groups and disease severities. *F. prausnitzii* and *Roseburia* species, the principal butyrate producers, are significantly reduced in both pediatric and adult asthma, with depletion severity correlating with disease control and exacerbation frequency (Zhang et al., 2022; Bradley and Haran, 2024). *Bacteroides* abundance shows variable associations: expansion in neutrophilic asthma has been reported in some cohorts, whereas reduction in Th2-high allergic phenotypes in others, suggesting endotype-specific microbial signatures (Machiels et al., 2014; Magne et al., 2020). *Akkermansia muciniphila* (*A. muciniphila*), a mucin-degrading specialist critical for barrier integrity, is consistently diminished in severe asthma and steroid-resistant subtypes (Machiels et al., 2014; Magne et al., 2020). Conversely, *Enterobacteriaceae* and other Gram-negative taxa expand in non-allergic neutrophilic asthma, elevating systemic LPS and reinforcing Th17 polarization (Furusawa et al., 2013; Mukhopadhya and Louis, 2025). These compositional shifts are not merely epiphenomena but functionally drive the immunopathological cascades described in Section 2.2.

### 3.2 SCFA-mediated immunoregulation

The three principal SCFAs (acetate, propionate, and butyrate) enter systemic circulation through the hepatic portal vein and regulate pulmonary immunity through distinct receptor-dependent and epigenetic mechanisms. Butyrate, the most potent HDAC inhibitor among SCFAs, suppresses NF-κB activation in airway epithelial cells and promotes FOXP3+ Treg differentiation through GPR43 engagement (Furusawa et al., 2013; Mukhopadhya and Louis, 2025). These effects synergize with the ILC2-suppressive and mast cell-inhibitory actions described in Section 2.1, creating a multi-layered anti-inflammatory network (Druszczynska et al., 2024). Propionate, generated by *Bacteroides* through succinate metabolism, attenuates allergic airway inflammation by dampening Th2 cytokine production and reducing eosinophilic infiltration (Jiang et al., 2023). Acetate, produced by *A. muciniphila* and diverse commensals, activates alveolar macrophage anti-inflammatory programs and enhances epithelial tight junction expression, thereby attenuating allergen penetration (Cani et al., 2022). The relative proportions of these SCFAs, rather than absolute concentrations, may determine immunological outcomes: butyrate-dominant profiles favor Treg/IL-10 responses, while acetate-enriched environments promote IL-22-mediated barrier repair (Konopelski and Mogilnicka, 2022; Zhuo et al., 2024).

### 3.3 Tryptophan metabolism and AhR signaling

Tryptophan-metabolizing bacteria, including *Lactobacillus reuteri* (*L. reuteri*) and *Clostridium sporogenes*, generate indole-3-propionic acid (IPA) and aryl hydrocarbon receptor (AhR) ligands that constitute a distinct class of pulmonary immunomodulators (Konopelski and Mogilnicka, 2022; Zhuo et al., 2024). These metabolites reach the lungs via systemic circulation, where they activate AhR signaling in pulmonary epithelial cells and ILCs (Zhang et al., 2024). AhR activation enhances epithelial barrier function by upregulating tight junction proteins and mucin production, while simultaneously modulating ILC2 and ILC3 responses to promote tissue repair and pathogen defense (Jin et al., 2026). In asthma, reduced tryptophan-metabolizing capacity correlates with increased airway hyperresponsiveness, suggesting that AhR pathway deficiency may contribute to disease severity (Lamas et al., 2018). Unlike SCFAs, which primarily suppress effector responses, AhR ligands appear to balance host defense with inflammatory resolution, positioning them as complementary rather than redundant immunomodulators.

### 3.4 Gut-to-lung transmission routes

Intestinal microbial signals reach the pulmonary compartment through three primary routes, each with distinct implications for asthma pathogenesis. First, metabolite-mediated systemic effects constitute the dominant pathway: SCFAs and indole derivatives enter the bloodstream through the hepatic portal vein, partially bypass hepatic first-pass metabolism, and modulate bone marrow hematopoiesis to generate lung-homing DC precursors and alternatively activated macrophages (Miao et al., 2024). Second, immune cell trafficking provides targeted delivery: intestinal Treg cells educated in gut-associated lymphoid tissues utilize the CCL25/CCR9 chemokine axis to seed pulmonary compartments and suppress inflammatory responses (Evans-Marin et al., 2015). Third, direct microbial translocation via lymphatic and blood circulation occurs when barrier dysfunction permits bacterial fragments or intact cells to be phagocytosed by DCs that migrate to pulmonary tissues; this pathway is particularly relevant in neutrophilic asthma, where LPS translocation amplifies TLR4 signaling and Th17 polarization (Thaiss et al., 2016). The relative contribution of each route varies by asthma endotype: Th2-high allergic asthma is predominantly metabolite-driven, while neutrophilic asthma involves greater microbial translocation and immune cell trafficking.

### 3.5 Lung-to-gut feedback in asthma

The pulmonary compartment reciprocally influences intestinal microbial composition through inflammatory mediator release and neural signaling, though evidence in asthma specifically remains limited. In acute asthma exacerbations, elevated systemic IL-6 and TNF-α may perturb gut barrier integrity and promote Gram-negative expansion, creating a self-amplifying inflammatory loop (Yang et al., 2020). Conversely, respiratory infections that trigger asthma exacerbations, such as rhinovirus and respiratory syncytial virus, have been shown to rapidly remodel distal intestinal ecosystems by reducing *Lactobacillus* and *Lactococcus* abundance while increasing *Enterobacteriaceae* (Yu et al., 2023). These observations suggest that asthma is not merely a recipient of gut-derived signals but an active participant in bidirectional axis dysregulation, though mechanistic studies distinguishing cause from effect are urgently needed.

In summary, the intestinal microbiota in asthma is characterized by depletion of butyrate producers and AhR ligand-generating taxa, accompanied by expansion of Gram-negative LPS sources. These compositional shifts functionally drive the endotype-specific immunopathological cascades described in Section 2 through metabolite-mediated, immune cell-dependent, and direct translocation pathways.

## 4 Gut microbiota in asthma

### 4.1 Gut dysbiosis contributes to asthma pathogenesis

**Figure 3:**
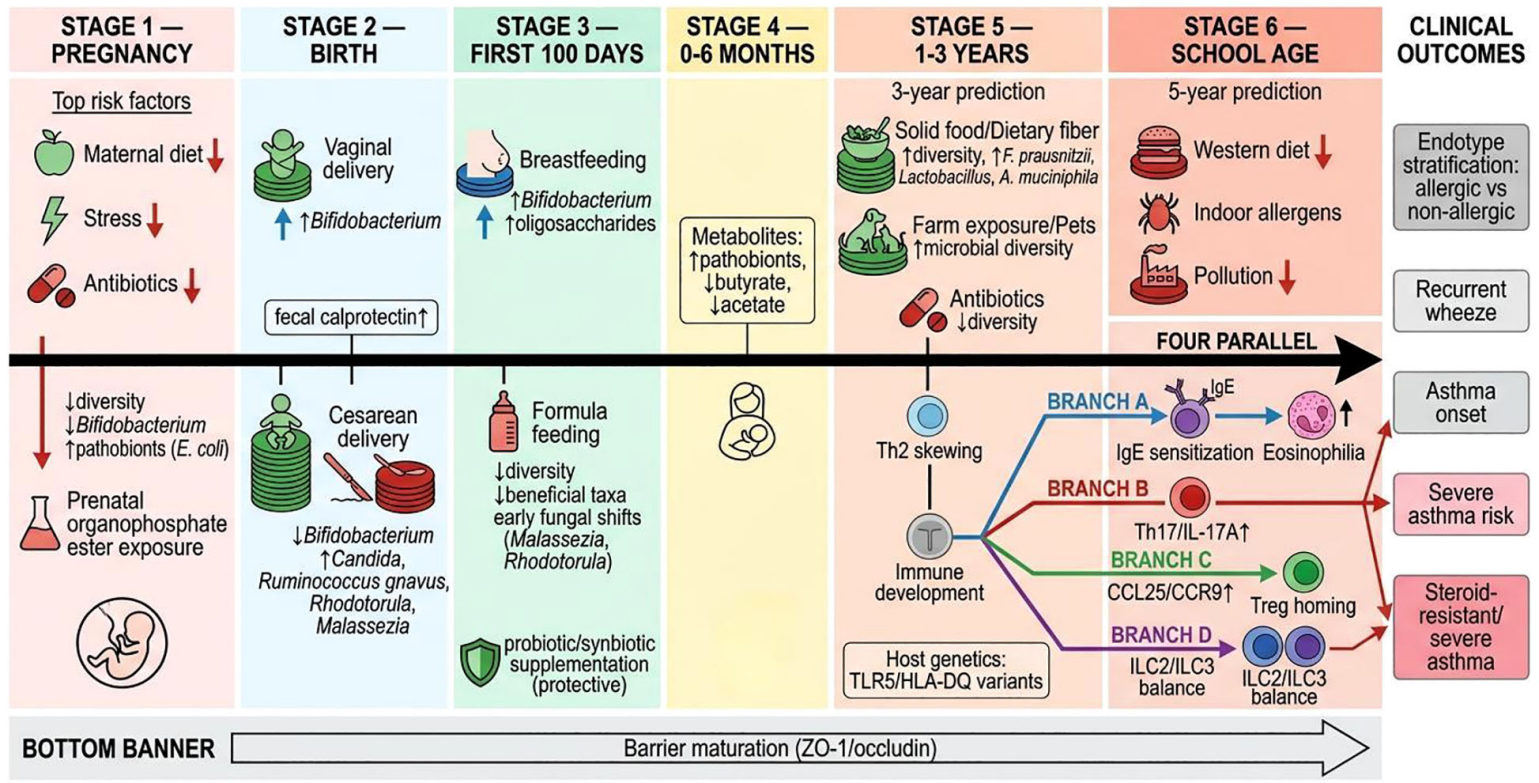
Developmental origins of asthma: critical windows of gut microbiome colonization and immune imprinting.

The intricate interplay between intestinal microbial communities and asthma development has garnered substantial attention (**Figure 3**). We classified all included studies published between 2011–2025 into three evidence tiers per evidence-based medicine standards: clinical observational studies, randomized controlled trials, and preclinical animal studies (**Table 1**). The associations between gut dysbiosis and asthma present distinct characteristics across different study types and asthma endotypes.

**Table 1:** Clinical studies on gut microbiota and asthma from 2011 to 2025.

Study type	Country	Sample	Detection technologies	Key findings	Reference
Prospective cohort study	Denmark	411 high-risk children, 6-year follow-up	16S rRNA, DGGE	Reduced intestinal flora diversity was associated with allergic rhinitis and increased peripheral blood eosinophils, but not with the development of asthma.	(Bisgaard et al., 2011)
	Sweden	47 high-risk infants, 7-year follow-up	16S rDNA	Reduced gut microbiota diversity as measured by 16S rDNA sequencing is associated with the development of asthma.	(Abrahamsson et al., 2014)
	Belgium	110 infants, 3-year follow-up	16S rRNA, DGGE	Increased abundance of Bacteroides fragilis subgroup and Clostridium cocleatum subgroup XIVa was associated with the development of asthma.	(Vael et al., 2011)
	USA	298 infants, 4-year follow-up	16S rRNA, metabolomics	Decreased abundance of Bifidobacterium, Akkermansia, and Faecalibacterium,increased abundance of Candida and Rhodotorula,and elevated pro-inflammatory metabolitesare associated with the development of asthma.	(Fujimura et al., 2016)
	Taiwan, China	44 infants, 3-year follow-up	16S rRNA, GGE	Increased abundances of Lachnospiraceae and Ruminococcus gnavus were associated with the development of asthma.	(Chua et al., 2018)
	Austria, Finland, France, Germany, and Switzerland	758 infants, 6-year follow-up	16S rRNA, ELISA	Decreased abundance of Escherichia coli and elevated fecal calprotectin were associated with the development of asthma.	(Orivuori et al., 2015)
	Canada	343 infants, 5-year follow-up	16S rRNA, ITS-2 rRNA	Decreased abundance of Malassezia, Debaryomyces, Candida,and increased abundance of Rhodotorula, Candida non-albicans, Cladosporium, Alternariaare associated with the development of asthma.	(Boutin et al., 2021)
	USA	152 children with food allergy, divided into asthma group and control group	Metagenomics	Decreased abundance of Bifidobacterium breve, Bifidobacterium catenulatum, Prevotella, Veillonella sp. CAG:933 and Bacteroides vulgatus,and increased abundance of three Bacteroides speciesare associated with the development of asthma.	(Mahdavinia et al., 2023)
	USA	24 adult asthma patients and 8 healthy controls	16S rRNA	A reduced Bacteroidetes/Firmicutes ratio is associated with the development of asthma.	(Begley et al., 2018)
Case-control study	UK	36 adult asthma patients and 185 healthy controls	Metagenomics	Decreased abundances of *F. prausnitzii*, Wardlawella and Bacteroides stercoris, increased abundances of Clostridium and Eggerthella lenta, as well as altered SCFA levels, were associated with the development of asthma.	(Okba et al., 2018)
	Egypt	80 adult asthma patients and 40 healthy controls	Semi-quantitative fecal culture	Reduced ratio of Bacteroidetes to Firmicutes is associated with asthma.	(Arrieta et al., 2018)
	Ecuador	27 asthmatic children and 70 healthy controls	16S rRNA, 18S rRNA, GC	Reduced abundance of Veillonella, Pichia kudriavzevii, and members of the order Saccharomycetales, along with elevated Bifidobacterium abundance, decreased fecal acetate levels, and increased fecal caproic acid levels, are associated with asthma.	(Wang et al., 2018)
	China	29 asthmatic patients and 15 healthy controls	16S rRNA	Elevated amino acid levels and increased abundance of *Clostridium*, *Veillonella*, and *Schaedlerella* are linked to asthma.	(Wang et al., 2021)
	China	37 asthmatic children and 20 healthy controls	16S rRNA, non-targeted metabolomics	Reduced abundance of *Lactococcus*, along with increased *Bacteroides* abundance and decreased levels of lipid and tryptophan metabolites, is associated with asthma.	(Zheng et al., 2022)
Cross-sectional study	Taiwan, China	35 asthmatic children, 28 children with allergic rhinitis, and 26 healthy controls	16S rRNA	Reduced abundance of Verrucomicrobia is linked to elevated *Lactococcus* abundance and asthma.	(Chiu et al., 2019)
	Turkey	92 asthmatic children and 88 healthy controls	DNA qPCR	Reduced abundance of mucin-degrading bacteria including *Akkermansia muciniphila* and *Parabacteroides* is associated with asthma.	(Demirci et al., 2019)
	Netherlands	1,440 children	16S rRNA	Reduced abundance of *Lachnospira*, *Coprococcus*, and *Christensenellaceae*, together with elevated *Dialister* abundance, is associated with asthma.	(Hu et al., 2021)
	China	23 asthmatic children, 18 children with allergic rhinitis, and 19 healthy controls	Metagenomics	Increased gut microbiota diversity and elevated *Bacteroides* and Verrucomicrobia abundance are associated with asthma.	(Wan et al., 2023)
	Korea	28 patients with eosinophilic chronic rhinosinusitis and 18 healthy controls	16S rRNA	Reduced abundance of *Lachnospira* and *Butyricimonas*, together with elevated *Prevotella* abundance, is associated with eosinophilic chronic rhinosinusitis and asthma comorbidity.	(Gu et al., 2023)
Nested case control study	Nordic	6,690 children	-	Antibiotic use during the prenatal period and in offspring after birth was associated with the development of asthma in children.	(Metsälä et al., 2015)
	China	332 eczema cases and 332 healthy controls	OPEs detection, gut microbiota sequencing	Increased gut microbiota α-diversity and decreased abundance of Lachnoclostridium were associated with the development of childhood asthma.	(Zhou et al., 2025)
	Canada	319 infants	16S rRNA, metabolomics	Decreased abundances of Lachnospira, Veillonella, Faecalibacterium and Roseburia, as well as reduced fecal acetate levels, were associated with the development of asthma.	(Arrieta et al., 2015)
	Austria, Finland, France, Germany, and Switzerland	930 infants, 6-year follow-up	16S rRNA, LC	Reduced abundance of *Rothia* and *Coprococcus*, along with decreased fecal butyrate levels, are associated with asthma.	(Depner et al., 2020)
Randomized controlled trial	USA	138 infants	16S rRNA, untargeted metabolomics	Increased abundance of Veillonella and elevated levels of metabolites in the histidine pathway are associated with asthma development and wheezing frequency.	(Lee-Sarwar et al., 2022)

*DGGE, denaturing gradient gel electrophoresis; GGE, gradient gel electrophoresis; ELISA, enzyme-linked immunosorbent assay; SCFA, short-chain fatty acid; ITS-2 rRNA, internal transcribed spacer 2 ribosomal RNA; GC, gas chromatography; OPE, organophosphate ester; LC, liquid chromatography.

(1) sample sizes (e.g., 411, 47, 110 participants); (2) follow-up durations (e.g., 3-year, 6-year); (3) detection technologies (e.g., 16S rRNA, metabolomics, metagenomics); and (4) publication years in the Reference column. All values are self-explanatory and consistent with standard clinical study reporting format.

#### 4.1.1 Clinical observational studies

Observational studies constitute the most abundant evidence tier, clarifying gut microbiota characteristics in asthmatic populations, identifying early-life risk factors and microbial biomarkers, though they cannot establish causal relationships between dysbiosis and asthma. Prospective cohort studies demonstrate that reduced early-life microbial diversity predicts subsequent asthma onset, with impairment of immune tolerance and metabolic homeostasis as mediating mechanisms. Danish researchers following 414 high-risk children observed reduced diversity correlating with allergic rhinitis and peripheral eosinophilia (Bisgaard et al., 2011), while Swedish cohorts confirmed this association (Abrahamsson et al., 2014). Belgian investigators identified *Enterococcus*/*Veillonella* enrichment as predictive of disease onset at 3-year follow-up (Vael et al., 2011), and Fujimura et al. (2016) linked reduced bacterial load and altered metabolic activity at 3 months to asthma development. Twin cohort studies associated *Ruminococcus gnavus* abundance with infant respiratory allergies (Chua et al., 2018), and a multinational European investigation identified elevated *Escherichia coli* and fecal calprotectin as predictors of asthma onset, implicating intestinal inflammation as a mediating mechanism (Orivuori et al., 2015). Early-life fungal community shifts also associate with inhalant atopy development by age 5 (Boutin et al., 2021). In older children and adults, *Bacteroides* and *Bifidobacterium* abundance differences correlate with asthma development (Mahdavinia et al., 2023), while adult cohorts confirm associations with aeroallergen sensitization and lung function (Begley et al., 2018).

Case-control studies across diverse populations consistently identify reduced Bacteroidetes/Firmicutes ratios as a unifying feature (Okba et al., 2018), with depleted *Bifidobacterium*, *Akkermansia*, and *Faecalibacterium* contrasting with elevated *Ruminococcaceae* and *Rhodospirillaceae* (Arrieta et al., 2018). British investigations noted diminished *Lachnospiraceae*, *Ruminococcaceae*, and *Christensenellaceae* alongside *Acinetobacter* expansion (Wang et al., 2018). Critically, microbial signatures vary by endotype: allergic asthma shows *Lactococcus* depletion and altered lipid metabolism (Zheng et al., 2022), whereas non-allergic asthma exhibits *Candidatus Accumulibacter* enrichment associated with histamine upregulation, and severe asthma shows *Veillonellaceae* and *Prevotellaceae* expansion compared with mild cases (Wang et al., 2021).

Cross-sectional and nested case-control analyses further reveal age-dependent patterns and metabolic perturbations. Pediatric populations show depletion of Firmicutes and mucin-degrading taxa such as *A. muciniphila* (Chiu et al., 2019; Demirci et al., 2019; Hu et al., 2021; Wan et al., 2023), while adult eosinophilic asthma exhibits reduced *Lachnospiraceae* and *Oscillospiraceae* alongside elevated Bacteroidetes (Gu et al., 2023). Large-sample nested studies verify these findings: a Nordic investigation of 6,690 participants linked reduced *Acidaminococcus* abundance and fecal butyrate concentrations to asthma (Metsälä et al., 2015), and a Chinese cohort identified prenatal organophosphate ester exposure as a risk factor via gut microbiota alteration (Zhou et al., 2025). Multiple studies collectively confirm depleted butyrate, propionate, and acetate in asthmatic patients (Arrieta et al., 2015; Depner et al., 2020).

#### 4.1.2 Randomized controlled trials

RCTs represent the gold standard for evaluating causal intervention effects, though current relevant trials remain limited in quantity and follow-up duration. A randomized trial of 138 infants demonstrated that maternal probiotic supplementation during pregnancy modified offspring intestinal microbiota composition (Lee-Sarwar et al., 2022); however, long-term protective efficacy against childhood asthma has not been fully verified due to insufficient follow-up until school age. This inconsistency between microbial changes and clinical outcomes indicates that simply altering gut microbiota composition cannot permanently reshape immune function, highlighting the need for mechanistically informed intervention designs and longer observation periods.

#### 4.1.3 Preclinical animal studies

Animal models provide irreplaceable tools for exploring molecular mechanisms linking gut dysbiosis and asthma, though their results require cautious extrapolation to human populations due to differences in microbial community composition, genetics, and environmental exposures. Relevant animal experiments are integrated in the mechanistic sections below and the FMT intervention section.

Collectively, all evidence tiers confirm that asthmatic patients exhibit diminished intestinal microbial diversity, altered composition, and disturbed metabolite profiles. A universal feature across populations is the depletion of *Bifidobacterium*, *Veillonella*, and *Lachnospira*, accompanied by expansion of *Clostridium* and *Rhodotorula*. These observational patterns establish the foundation for mechanistic investigations but do not establish causality; the following sections dissect how these compositional shifts functionally drive asthma pathogenesis.

### 4.2 Mechanistic pathways of intestinal dysbiosis in asthma development

**Figure 4:**
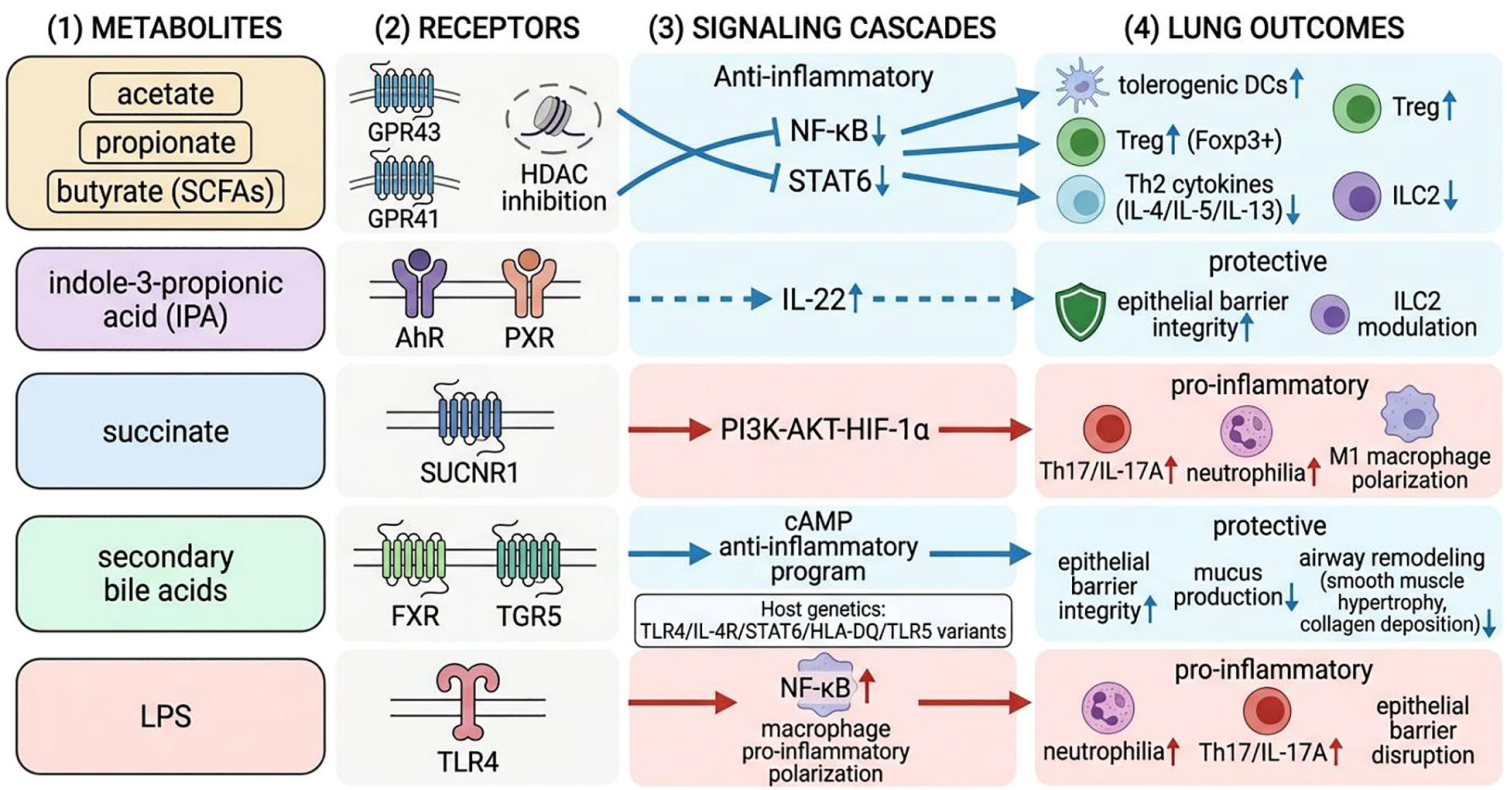
Microbial metabolites and immune pathways linking gut dysbiosis to asthma.

The specific mechanisms by which intestinal dysbiosis participates in asthma pathogenesis remain incompletely elucidated and are not identical across endotypes. Th2-high allergic asthma appears predominantly driven by SCFA-mediated Treg/ILC2 imbalance and IgE class switching, whereas non-Th2 variants, including neutrophilic and obesity-associated asthma, involve LPS translocation-induced TLR4 activation and IL-17-driven neutrophilic recruitment via distinct microbial triggers. Existing evidence suggests that intestinal dysbiosis influences pulmonary immune homeostasis through three primary pathways: immune cell functional regulation, microbial metabolite signaling transduction, and disruption of intestinal barrier integrity. These mechanisms collectively constitute the molecular basis of bidirectional gut-lung axis communication (see **Figure 4**).

#### 4.2.1 Immune cell modulation: the core cellular mechanism of gut-lung axis

Intestinal microbiota profoundly shapes pulmonary innate and adaptive immune cell function, with distinct cellular targets across asthma endotypes. Alveolar macrophage polarization represents a major target of gut-derived signals: intestinal dysbiosis elevates microbiota-derived succinate in lung tissue, driving M1 macrophage polarization via SUCNR1-dependent PI3K/AKT/HIF-1α activation and promoting pro-inflammatory cytokine release (Wang et al., 2023). The composition of the gut microbiota modulates DC immunogenicity, with higher bacterial diversity correlating with altered costimulatory molecule expression and modified IL-12/IL-10 production ratios in monocyte-derived DCs (Negi et al., 2019).

ILCs constitute another critical interface. Intestinal ILC2s maintain homeostasis through IL-10 production, constraining ILC3 and Th17 expansion; selective ILC2 loss leads to compensatory ILC3 accumulation and heightened IL-17-driven pathology (Guo et al., 2023). Pharmacological targeting of RORγt in ILC3s attenuates this cascade, demonstrating therapeutic potential through selective ILC manipulation (Sudworth et al., 2022).

Adaptive immunity is similarly regulated through gut-lung trafficking. Gut commensals prime DCs to orchestrate Th1/Th2/Th17/Treg differentiation, with effector subsets subsequently migrating to respiratory tissues via chemokine-guided pathways (Li et al., 2025). Allergic asthma is characterized by Th2-skewed responses driven by IL-4/IL-13/STAT6 signaling, whereas non-allergic and obesity-associated phenotypes demonstrate Th17-predominant or mixed granulocytic profiles. Treg cells educated in intestinal lymphoid organs utilize the CCL25/CCR9 axis to seed the lungs and suppress inflammatory responses (Wang et al., 2024); disruption of this pathway in dysbiosis correlates with diminished pulmonary Treg frequencies. Th17 cells generated in response to intestinal bacterial signals promote neutrophilic inflammation when recruited to pulmonary compartments, with elevated Th17/Treg ratios marking severe asthma phenotypes (Wu et al., 2022). Beyond T cells, gut microbes shape systemic humoral immunity by directing plasma cell differentiation and IgA production in gut-associated lymphoid tissues (Bertrand et al., 2023); high-IgE states are associated with altered intestinal bacterial composition, particularly reduced *Clostridium* and expanded *Lactobacillus* populations (Bertrand et al., 2023). These perturbations permissively influence IgE-mediated responses at distal mucosal sites, as evidenced by enhanced airway inflammation following intestinal fungal dysbiosis in the absence of direct lung colonization (Christodoulou and Clarke, 2024).

#### 4.2.2 Microbial metabolites: key signaling molecules

SCFAs, particularly butyrate produced by *Clostridiaceae* and related Firmicutes taxa, function as critical regulators of pulmonary immune homeostasis through dual mechanisms involving HDAC inhibition and GPCR activation (Mukhopadhya and Louis, 2025). Butyrate suppresses inflammatory ILC2 proliferation and GATA3 expression, while promoting tolerogenic DC phenotypes, with pulmonary effects contingent on concentration-dependent GPR43 and GPR41 engagement (Corrêa et al., 2022; Jiang et al., 2023). Propionate shares overlapping yet distinct signaling properties, and both metabolites coordinately suppress NF-κB and STAT6 pathways to attenuate allergic lung diseases (Jiang et al., 2023).

The immunomodulatory repertoire extends beyond SCFAs to tryptophan catabolites. Bacterial conversion generates IPA and related derivatives that engage AhR and PXR to enhance intestinal barrier integrity and modulate systemic immunity (Konopelski and Mogilnicka, 2022). IPA produced by Clostridium sporogenes and *L. reuteri* promotes tolerogenic DC differentiation and Treg function while exerting direct antioxidant effects on airway epithelial cells through ROS scavenging and suppression of TNF-α and IL-6 (Zhuo et al., 2024).

Bile acid metabolites constitute an emerging class of gut-lung signaling molecules. Microbial transformation of primary bile acids generates secondary metabolites that activate FXR and GPBAR1 signaling axes with relevance for pulmonary inflammation (Caparrós-Martín et al., 2023). FXR activation modulates macrophage polarization while GPBAR1 triggers cAMP-dependent anti-inflammatory programs, though concentration-dependent effects and paradoxical fibrogenic responses in certain contexts necessitate careful therapeutic consideration.

#### 4.2.3 Gut barrier dysfunction: a gateway for systemic inflammation in asthma

Intestinal epithelial integrity rests upon tight junction complexes, with ZO-1 and occludin forming the core structures that seal the paracellular space (Gieryńska et al., 2022). Microbial dysbiosis disrupts this barrier as Gram-negative bacterial overgrowth increases luminal LPS, which upon binding TLR4 on enterocytes triggers MLCK activation (Matar et al., 2024). This kinase phosphorylates the myosin regulatory light chain, inducing cytoskeletal contraction that opens tight junctions and permits microbial product translocation.

LPS translocation initiates systemic inflammation, as circulating monocytes encountering bacterial products release IL-6 and IL-17, which then travel to the lungs (Shen et al., 2023). IL-6 promotes Th2 and Th17 differentiation, while IL-17 recruits neutrophils and synergizes with TGF-β to produce fibrogenic effects (Sun et al., 2023). Structural cells respond directly: fibroblasts activate into myofibroblasts, proliferate, and migrate, leading to collagen deposition and accumulation of types I, III, and V collagen alongside tenascin and fibronectin (Tiotiu, 2025). Concurrently, airway smooth muscle cells hypertrophy, narrowing the lumen. TGF-β orchestrates much of this remodeling, released by eosinophils and injured epithelial cells, with IL-11 and IL-13 amplifying the response to produce subepithelial fibrosis (Tiotiu et al., 2025). Wall thickening correlates with disease severity, and these structural changes are largely irreversible.

Epithelial injury at both intestinal and pulmonary sites perpetuates this cycle, as damaged airway epithelium releases alarmins including TSLP, IL-25, and IL-33, which activate ILCs and compromise tight junctions, thereby increasing allergen penetration and subsequent sensitization (Hong et al., 2020). The gut-lung axis operates through these shared molecular mechanisms, with barrier dysfunction propagating inflammation that further damages barriers in a self-amplifying loop.

#### 4.2.4 Microbe-host genetic interaction: determinants of asthma susceptibility

The interplay between host genetic architecture and microbial colonization patterns establishes a critical axis governing individual susceptibility to allergic airway disease. Genetic variants encoding pattern recognition receptors and cytokine signaling molecules sculpt the immunological landscape through which microbial signals are interpreted, creating distinct trajectories of inflammatory responsiveness.

TLR4 represents a pivotal gateway for host-microbe communication, recognizing LPS from Gram-negative bacteria (Wang et al., 2023). In allergic asthma models, OVA-LPS challenge activates the TLR4/NF-κB cascade, driving neutrophilic inflammation and Th2 cytokine production (Wu et al., 2022). Probiotic intervention attenuates this pathway by suppressing *Tlr4* expression and downstream MyD88 and NF-κB mediators, establishing that gut microbial manipulation can modulate TLR4-dependent pulmonary inflammation (Wu et al., 2022). Genetic variation in TLR4 further illustrates this interaction: individuals harboring hyporesponsive TLR4 variants exhibit altered gut microbiome composition, particularly reduced flagellated bacteria, impairing DC maturation and Th1-polarizing capacity (Fukaya et al., 2023).

The IL-4/IL-13/STAT6 axis constitutes a fundamental determinant of allergic inflammation. Liu et al (Liu et al., 2017). established that IL-4 receptor engagement suppresses airway inflammation by inhibiting STAT6 phosphorylation, with attenuated eosinophilic infiltration and diminished Th2 cytokine synthesis. These findings position STAT6 as a convergence point where microbial signals and host genetic factors integrate to regulate Th2 polarization. Genetic polymorphisms affecting IL-4Rα function would alter the threshold for microbial-triggered allergic sensitization, potentially reshaping the microbial niche through immunological selection pressures (Girdhar et al., 2025).

Class II major histocompatibility complex molecules present antigenic peptides to CD4+ T cells, with specific alleles conferring susceptibility to mold-sensitive asthma. Knutsen et al. (2010) found that HLA-DQB1*03* occurs at reduced frequency among children with moderate-to-severe asthma sensitized to *Alternaria alternata*. This allele appears protective against Th2-mediated inflammation, as HLA-DQB103-negative individuals exhibited exaggerated IL-5 and IL-13 synthesis upon fungal stimulation. This genetic variation modulates how fungal commensals trigger pathological responses, and the gut mycobiome serves as the primary reservoir for such fungal exposure during early immune education.

Toll-like receptor 5 (TLR5) polymorphisms provide compelling evidence for microbe-gene interaction. The rs5744168 variant, introducing a premature stop codon and generating a dominant-negative decoy receptor, associates with reduced asthma symptom burden (Whitehead et al., 2020). Bacterial flagellin, a TLR5 ligand enriched in house dust from asthma patients, exacerbates airway inflammation in murine models, with co-exposure synergistically amplifying eosinophilic and neutrophilic infiltration (Whitehead et al., 2020). Individuals with functional TLR5 deficiency are shielded from these proinflammatory consequences, likely because this genetic variant alters gut microbiome composition and dampens DC activation. Collectively, TLR5 polymorphisms modulate asthma risk through dual mechanisms: direct attenuation of flagellin-mediated pulmonary inflammation and indirect remodeling of gut microbial communities that shape systemic immune priming.

At the cellular level, these genetic variants converge on DC function as a critical node integrating microbial and host signals. TLR5 activation promotes maturation and cytokine secretion, while HLA-DQ variants alter peptide-presentation efficiency and IL-4R polymorphisms modulate the cytokine milieu, thereby influencing DC-T cell crosstalk. The gastrointestinal microbiome serves as the primary source of immunomodulatory signals that engage these genetic pathways, with host variation creating individualized response patterns to shared microbial communities. Asthma susceptibility thus emerges from a dynamic interplay between inherited genetic variants and acquired patterns of microbial colonization, a concept that directly informs the design of precision microbiome-based interventions tailored to host genotype.

## 5 Potential therapeutic strategies based on gut microbiota

The mechanistic pathways delineated in Section 4 establish multiple actionable targets for microbiota-directed interventions. This section critically evaluates the translational evidence for strategies that modulate these pathways, organized by intervention intensity from supplementation to replacement.

### 5.1 Probiotics, postbiotics, and prebiotics

The bidirectional communication between gut microbiota and respiratory diseases, termed the gut-lung axis, has opened novel therapeutic avenues for asthma management that extend beyond conventional pharmacological interventions. Within this framework, microbiota-targeted strategies are transitioning from theoretical concepts to clinical practice, establishing a multi-tiered intervention system comprising probiotics, postbiotics, and prebiotics (Ji et al., 2023). Recent years have witnessed substantial advances in the clinical application of these three microbiota-directed therapies (**Table 2**).

**Table 2:** Clinical studies on probiotics, postbiotics or prebiotics for asthma prevention and treatment from 2001 to 2025.

Type	Treatment	Key findings	Reference
Probiotics	Pregnant women were randomly assigned to receive either placebo or *Lactobacillus rhamnosus* for 2–4 weeks; their newborns continued the intervention for 6 months after delivery, with follow-up for 2 years.	*Lactobacillus rhamnosus* prevented the onset of asthma.	(Kalliomäki et al., 2001)
	Pregnant women at high risk of allergy received either placebo or a probiotic mixture (2 strains of *Lactobacillus*, *Bifidobacterium*, and *Propionibacterium*) for 1 month; their newborns continued to receive placebo or probiotics after delivery, along with galacto-oligosaccharides for 6 months, with follow-up for 5 years.	The probiotic mixture prevented asthma in children born via cesarean section, but prevented the development of airway inflammation in children.	(Kuitunen et al., 2009)
	Pregnant women at high risk of allergy received either placebo or a probiotic mixture (*Lactobacillus rhamnosus*, *Bifidobacterium breve*, *Propionibacterium freudenreichii*) for 1 month; their newborns continued to receive placebo or probiotics after delivery, along with galacto-oligosaccharides for 6 months, with follow-up for 2 years.	The probiotic mixture prevented the onset of asthma.	(Kukkonen et al., 2010)
	Pregnant women were randomly assigned to receive either placebo or *Lactobacillus royi* for 1 month; their newborns continued the intervention for 1 year after delivery, with follow-up for 7 years.	In the long term, probiotics could not prevent the onset of asthma.	(Abrahamsson et al., 2013)
	Asthma patients received either placebo or *Lactobacillus rhamnosus* for 5.5 months.	*Lactobacillus rhamnosus* could not alleviate symptoms in asthma patients.	(Helin et al., 2002)
	Asthmatic children received either placebo or a probiotic (*Bifidobacterium longum*, *Bifidobacterium infantis*, *Bifidobacterium breve*) for 1 month.	The probiotic alleviated symptoms in asthmatic children.	(Miraglia Del Giudice et al., 2017)
	Asthma patients received either placebo or *Bifidobacterium lactis* for 3 months.	The probiotic alleviated symptoms in asthma patients.	(Liu et al., 2021)
	Asthma patients received either placebo or a probiotic mixture (*Lactobacillus reuteri*, *Bifidobacterium breve*) for 2 months.	*Lactobacillus reuteri* and *Bifidobacterium breve* reduced the frequency of acute asthma exacerbations.	(Drago et al., 2022)
Postbiotics	Preschool children with recurrent wheezing received OM-85 BV or placebo, with follow-up for 12 months.	OM-85 BV reduced wheezing attack rate and shortened attack duration by 2 days.	(Razi et al., 2010)
	Children with asthma received conventional treatment with or without OM-85 BV, compared with ICS group.	OM-85 BV reduced respiratory infections and asthma attacks.	(Lu et al., 2015)
	Infants with post-bronchiolitis asthma; received conventional treatment with or without OM-85 BV, with follow-up for 1 year.	OM-85 BV decreased attack frequency and duration and alleviate inflammation.	(Han et al., 2016)
	Children with allergic asthma received PMBL or placebo, with follow-up for 9 months.	PMBL reduced asthma exacerbation frequency and days, and prolonged intervals between exacerbations.	(Emeryk et al., 2018)
	Children with allergic asthma and dust mite allergy received PMBL or placebo sublingually for 12 weeks.	PMBL enhanced immunoregulatory function.	(Bartkowiak-Emeryk et al., 2021)
	Children with asthma received conventional treatment with or without Qipian, after propensity score matching.	Qipian improved asthma control, reduced exacerbation risk and rescue medication use.	(Li et al., 2022)
Prebiotics	Asthma patients or healthy controls received either placebo or galacto-oligosaccharides for 3 weeks; after a 2-week washout period, they crossed over to receive placebo or prebiotics for 3 weeks.	Galacto-oligosaccharides reduced airway hyperresponsiveness and airway inflammation.	(Williams et al., 2016)
	Adult asthma patients received placebo, soluble fibre, or soluble fibre with probiotic intervention for 7 days.	Soluble fibre supplementation improved airway inflammation and asthma control, and modulated gut microbiome composition.	(McLoughlin et al., 2019)
	Patients with asthma and house dust mite allergy received either placebo or synbiotics for 4 weeks.	Synbiotics treatment could not relieve bronchial inflammation or late asthmatic response, but significantly enhanced peak expiratory flow in adult allergic asthma patients.	(van de Pol et al., 2011)

*PMBL, polyvalent mechanical bacterial lysate.

#### 5.1.1 Probiotics

Preclinical animal studies establish the mechanistic foundation for probiotic immunomodulation. Live microorganisms remodel mucosal immunity by boosting anti-inflammatory IL-10, dampening pro-inflammatory signaling, and driving Foxp3+ Treg induction alongside Th1 polarization (Furusawa et al., 2013; Mazziotta et al., 2023). In murine asthma models, oral administration of *L. reuteri*, *Lactobacillus rhamnosus* (*L. rhamnosus*), or *Bifidobacterium breve* (*B. breve*) attenuates airway inflammation, reduces inflammatory infiltrates, and ameliorates pulmonary pathology (Barbieri et al., 2017). *L. rhamnosus* CRL1505 further illustrates this potency by eliciting marked increases in Th1 and Th2 cytokines, fortifying systemic immune competence (Barbieri et al., 2017). These animal studies establish biological rationale but do not guarantee clinical translatability.

Clinical trials have generated encouraging yet inconsistent signals. Preventive RCTs examining maternal supplementation during pregnancy illustrate this complexity: Kalliomäki et al. (2001) reported protective efficacy with *L. rhamnosus* GG, whereas Abrahamsson et al. (2013) found no effect at 7-year follow-up using a different *Lactobacillus* strain. These divergent outcomes likely reflect strain-specific immunomodulatory capacities: *L. rhamnosus* GG exhibits robust TLR2-dependent IL-10 induction that other strains lack. The cesarean-specific protection observed by Kuitunen et al (Kuitunen et al., 2009; Kukkonen et al., 2010). further indicates that intervention efficacy is contingent on baseline microbial status; vaginally delivered infants already acquire maternal microbiota, leaving less room for probiotic-mediated modification. The negative outcome reported by Helin et al. (2002) with *L. rhamnosus* in birch-pollen allergic adolescents may reflect the timing of intervention relative to established sensitization; once IgE-mediated inflammation is entrenched, microbial modulation alone may be insufficient to reverse pathophysiology.

Therapeutic RCTs in established asthma present even greater interpretive challenges. Symptomatic benefits observed with multi-strain *Bifidobacterium* formulations (Miraglia Del Giudice et al., 2017; Liu et al., 2021; Drago et al., 2022) may operate through metabolite-mediated pathways rather than direct immune cell modulation, though mechanistic stratification was not performed in these trials. The heterogeneity across RCT outcomes reflects genuine biological and methodological sources of variation, including strain selection, disease stage at intervention, and baseline microbiome configuration. These discrepancies collectively argue against universal probiotic recommendations and instead support precision approaches matched to endotype and individual microbiome status.

#### 5.1.2 Postbiotics

Unlike live probiotics, postbiotics represent inanimate microbial entities or their components that confer host benefit (Vinderola et al., 2022). This category spans structural elements and metabolites, offering inherent stability, favorable safety margins, exact dosing, and streamlined logistics (Wei et al., 2024). Bacterial lysates, the most extensively studied postbiotic preparations, remodel mucosal immunity through distinct pathways.

Clinical observational studies have provided early signals of postbiotic efficacy in real-world settings. A retrospective cohort study of 795 asthmatic patients with propensity score matching demonstrated that polyvalent bacterial lysate Qipian combination therapy achieved asthma control rates of 92.4% at 3 months and 88.5% at 6 months, substantially exceeding control group rates of 86.3% and 84.5%, alongside significant decreases in cumulative short-acting β-agonist dosage and antimicrobial prescriptions (Li et al., 2022). While these findings suggest clinical utility, the retrospective design introduces inherent limitations: propensity score matching cannot fully account for unmeasured confounders such as disease severity gradients or physician prescribing preferences. The absence of mechanistic stratification further obscures whether benefits reflect genuine immunomodulation or merely reduced respiratory infection incidence.

Randomized controlled trials have generated the most rigorous evidence, though with notable heterogeneity in design and outcomes. A randomized double-blind trial enrolled 75 preschool children with recurrent wheezing; 12 months of OM-85 BV intervention reduced wheezing attack rates by 37.9% and shortened each episode duration by 2 days, with efficacy attributable to marked decreases in acute respiratory tract infections (Razi et al., 2010). The immunomodulatory mechanisms were further elucidated by Lu et al. (2015) in 60 asthmatic patients who received OM-85 BV, in combination with conventional inhaled corticosteroids. Peripheral blood natural killer T cell proportions increased alongside elevated IFN-γ/IL-4 ratios and IL-10 levels, while reductions in respiratory infections and antibiotic use surpassed those achieved with corticosteroid monotherapy. In 136 infants with post-bronchiolitis asthma, 3 months of OM-85 BV combination therapy significantly reduced IL-17 and IL-4 while increasing IL-10 and IFN-γ, downregulated α7 nicotinic acetylcholine receptor expression, and markedly improved wheezing frequency and duration (Han et al., 2016). Polyvalent mechanical bacterial lysate (PMBL) has similarly proven effective in allergic asthma. The EOLIA study enrolled 152 patients aged 6–16 years with allergic asthma, in which three months of sublingual PMBL administration reduced the frequency of asthma exacerbations (Emeryk et al., 2018). Detailed immunological analyses revealed that after 12 weeks of treatment in 49 patients, total T lymphocyte counts rose with immune restoration toward homeostasis (Bartkowiak-Emeryk et al., 2021).

Collectively, these findings indicate that bacterial-derived postbiotic preparations activate innate and adaptive immunity, enhance Treg function, restore Th1/Th2 balance, and offer safe effective non-pharmacological options for asthma prevention and acute exacerbation control. However, the reliance on combination therapy in most trials precludes isolation of postbiotic-specific effects, and the optimal timing, dosing, and strain composition for standalone postbiotic therapy remain undefined.

#### 5.1.3 Prebiotics

Prebiotics selectively nourish beneficial taxa and amplify SCFA output. Inulin, fructooligosaccharides, and galacto-oligosaccharides yield 85.55 mmol/L SCFAs at optimal ratios (1.33%: 2.00%: 2.67%), producing acetate, propionate, and butyrate that sustain intestinal integrity and activate free fatty acid receptors to recalibrate immune tone and quell airway inflammation (Kaewarsar et al., 2023).

Clinical evidence supports these mechanistic premises, though the evidence base remains preliminary. Synbiotic formulations combining probiotics with galacto-oligosaccharides yielded modest improvements in airway inflammation parameters compared with probiotic monotherapy, and short-course synbiotic administration reduced airway hyperresponsiveness in both asthmatic patients and healthy controls (Williams et al., 2016). A randomized double-blind three-way crossover trial in 17 stable asthma patients found that 7 days of inulin at 12 g/day failed to elevate plasma SCFA levels, yet significantly improved asthma control scores while decreasing sputum eosinophil proportions and suppressing *Hdac9* gene expression, with concurrent shifts in specific gut microbiota operational taxonomic units (McLoughlin et al., 2019). These findings suggest that prebiotic effects may operate through local colonic mechanisms or alternative pathways independent of circulating metabolite levels. A slightly larger trial with 29 house dust mite allergic asthmatic patients receiving 4 weeks of synbiotic treatment observed that allergen-induced serum IL-5 increase was constrained, and Th2 cytokine secretion from peripheral blood mononuclear cells was likewise curbed (van de Pol et al., 2011).

The heterogeneity across these early trials (small sample sizes, brief intervention periods, and lack of mechanistic stratification) precludes definitive conclusions. However, the rapid onset of symptomatic benefits in some studies suggests that prebiotics may offer adjunctive value, particularly in patients with demonstrable SCFA deficiency or compromised barrier function.

### 5.2 Fecal microbiota transplantation

Probiotic formulations and FMT both exert therapeutic effects by correcting intestinal dysbiosis. The former, however, exhibits substantial limitations. The human gut harbors approximately 100 trillion microorganisms. Orally administered probiotics typically fall three to four orders of magnitude below this scale (Sarita et al., 2024). Gastric acid and small intestinal transit further compromise viability. Compounding these quantitative and survival deficits, probiotics cultured *in vitro* frequently lose adaptive capacity for the complex physiological environment encountered *in vivo* (Petrariu et al., 2023). FMT circumvents these constraints. It delivers intact, stable microbial communities directly via enema, nasoduodenal tube, or colonoscopy (Lee et al., 2024). This approach achieves genuine restoration or replacement of damaged ecosystems.

Given the established centrality of gut microbiota perturbations in asthma pathogenesis, investigators have begun exploring FMT in animal models of allergic airway disease (**Table 3**). Fecal transplantation from mice orally administered *Saccharomyces boulardii* to OVA-induced asthmatic mice ameliorated inflammation and oxidative stress while attenuating pulmonary damage (Liu et al., 2024). FMT from healthy rats significantly improved lung function, reduced airway inflammation and collagen deposition, and restored intestinal SCFA levels in asthmatic recipients (Lai et al., 2025). Conversely, Moya Uribe et al. (2025) transplanted fecal material from asthmatic infants, non-asthmatic infants, healthy adults, and healthy mice into germ-free mice, resulting in baseline lung function impairment in all human microbiota recipients, with distinct allergic inflammatory responses arising from different microbial sources. This demonstration that gut microbiota structure independently compromises pulmonary function and enhances allergic susceptibility establishes a theoretical foundation for FMT as an asthma therapeutic strategy.

**Table 3:** Animal experiments on fecal microbiota transplantation for asthma from 2021 to 2025.

Fecal microbiota donor	Animal model	Key findings	Reference
*Saccharomyces boulardii*-treated mice	OVA-induced allergic asthma mouse model	Gut microbiota mediated the up-regulation of METTL3 by *Saccharomyces boulardii*, which in turn improved inflammation, oxidative stress and alleviated lung injury in asthmatic mice.	(Liu et al., 2024)
Healthy control rats or asthmatic rats	OVA-induced asthma rat model	Fecal microbiota from healthy donors improved airway inflammation and corrected short-chain fatty acid disturbances in asthmatic rats, likely via restoring intestinal flora and promoting short-chain fatty acid recovery	(Lai et al., 2025)
Asthmatic infants, non-asthmatic infants, healthy adults or healthy mice	Germ-free C57BL/6 mice	Mouse microbiota harbored more anti-inflammatory and allergy-antagonistic taxa; mice showed better baseline lung function and milder inflammatory responses after HDM exposure compared with human microbiota groups.	(Moya Uribe et al., 2025)

*OVA, ovalbumin; METTL3, methyltransferase-like 3; HDM, house dust mite; Th2, T helper 2 cells; IgE, immunoglobulin E; IL-4, interleukin-4; IL-5, interleukin-5; IFN-γ, interferon-γ.

Nevertheless, therapeutic efficacy remains contingent upon donor microbial configuration, recipient disease status, and timing of administration. Future research must refine transplantation protocols, prioritize identification of key functional strains, and elucidate precise mechanistic pathways to facilitate clinical translation. The invasive nature of FMT and the potential for adverse events, including pathogen transmission and metabolic syndrome transfer, necessitate rigorous donor screening and long-term safety monitoring before clinical implementation in asthma.

### 5.3 Dietary intervention

High-fiber diets and the Mediterranean diet represent upstream, non-pharmacological strategies for modulating the gut-lung axis through sustained dietary modification. High-fiber intake curtails pulmonary inflammation by amplifying SCFA production via reconfiguration of intestinal microbiota and elevation of beneficial taxa including *Bifidobacterium*, with subsequent GPR engagement dampening inflammatory cell activation and reducing IL-5 and IL-13 production from ILC2s (Lewis et al., 2019; Tang et al., 2024). The Mediterranean diet offers a convergent yet distinct pathway: its abundance of polyphenols and omega-3 fatty acids from plant-derived constituents and extra virgin olive oil fosters expansion of *Bifidobacterium*, *F. prausnitzii*, and *Roseburia* species to fortify intestinal barrier integrity and attenuate systemic inflammatory tone (Watanabe and Tatsuno, 2020).

Epidemiological evidence supports these laboratory findings, with multiple cohort studies and meta-analyses demonstrating significant inverse associations between Mediterranean diet adherence and adult asthma incidence, particularly with adequate consumption of olive oil, fish, and other cornerstone components (Huang et al., 2024). Though these dietary strategies diverge in implementation: fiber interventions directly feed microbial fermentation machinery, while the Mediterranean diet operates through broader phytochemical and lipid profiles, both ultimately harness SCFA-mediated pathways to modulate the gut-lung axis. The translational challenge lies in achieving sustained dietary adherence and identifying the specific microbial taxa and metabolite thresholds required for clinical benefit in distinct asthma endotypes.

## 6 Challenges and future perspectives

### 6.1 Methodological and translational limitations

Current evidence linking gut microbiota to asthma pathogenesis rests heavily on observational associations and small-scale intervention studies with limited follow-up duration (Hilton Boon et al., 2022). While metabolites such as SCFAs and tryptophan derivatives have emerged as critical mediators operating through GPCRs and AhR pathways (He et al., 2020), substantial gaps persist regarding their spatiotemporal dynamics, concentration-dependent effects, and cell-type specific actions. Butyrate exemplifies this complexity: at physiological concentrations, it fortifies epithelial barrier integrity and promotes Treg differentiation via HDAC inhibition (Eshleman et al., 2024), yet elevated levels paradoxically compromise tight junction function in intestinal monolayers (Zuo et al., 2020). The molecular switches governing such biphasic responses await elucidation. Future investigations must leverage single-cell transcriptomics, spatial metabolomics, and organoid-based co-culture systems to construct high-resolution maps of microbe-host signaling networks across anatomical compartments, with particular attention to dose-response relationships and tissue-specific thresholds.

### 6.2 Heterogeneity and precision implementation

Asthma patients exhibit remarkable interindividual variability in gut microbiota composition, shaped by genetic architecture, early-life exposures, dietary patterns, and geographic context (Cheng et al., 2023). This variability manifests in inconsistent microbial signatures across cohorts: some investigations highlight depletion of *Lachnospira* and *Faecalibacterium*, while others emphasize expansion of *Ruminococcaceae* or *Rhodospirillaceae* (Zou et al., 2021). Such discrepancies likely reflect distinct asthma endotypes, each characterized by unique immunological trajectories and therapeutic responsiveness (Huang, 2025). The notion of universal probiotic formulations or standardized dietary interventions appears increasingly untenable. Precision microbiome medicine demands stratification frameworks integrating host genotype, metabolomic profiles, and immune phenotypes (Tegegne and Savidge, 2025). Polymorphisms in pattern recognition receptors such as TLR4 and TLR5 fundamentally alter how individuals process microbial signals (Chen et al., 2025), suggesting that genetic screening could guide targeted interventions. Adaptive clinical trial designs that match specific microbial deficits or excesses with corresponding corrective strategies will be required (Kaizer et al., 2023), moving beyond one-size-fits-all approaches toward bespoke therapeutic regimens.

### 6.3 Standardization and safety in clinical translation

The translational pipeline from mechanistic insight to clinical practice remains strikingly underdeveloped. Randomized controlled trials of probiotic supplementation during pregnancy have successfully modified offspring microbiota composition, yet definitive protection against childhood asthma remains unproven (Xiao et al., 2025). FMT demonstrates promising effects in animal models, but standardization of donor screening, preparation protocols, and delivery routes presents formidable challenges (Li et al., 2017). Safety considerations loom large, particularly regarding immunocompromised populations where translocation of opportunistic organisms poses genuine risks (Hayek et al., 2025). The field urgently requires large-scale, multicenter trials with hard clinical endpoints, extended observation periods, and robust biomarker stratification to establish both efficacy and safety profiles of microbiota-targeted therapies in diverse asthma phenotypes (Buccheri et al., 2025). Only through such rigorous validation can we determine whether the gut-lung axis represents a viable therapeutic target or merely an epiphenomenon of disease pathophysiology.

### 6.4 Strategic priorities for advancing the field

Realizing the therapeutic potential of the gut-lung axis in asthma demands a decisive paradigm shift from correlation to causation, from descriptive association to mechanistic understanding. Longitudinal cohort designs tracking subjects from prenatal periods through childhood will capture critical developmental windows wherein microbial colonization patterns imprint durable immune trajectories (Wang et al., 2026). Interventional studies should embrace precision methodologies: metabolite-engineered probiotics, targeted bacteriophage therapies, and synthetic ecology approaches offer greater specificity than traditional supplementation (Wang et al., 2025). Artificial intelligence and machine learning algorithms applied to multi-dimensional datasets promise to identify subtle patterns invisible to conventional analysis, enabling predictive modeling of disease risk and treatment response based on individual microbial fingerprints, genetic susceptibility, and environmental context (Amen et al., 2025). The ultimate vision encompasses individualized microbiome medicine wherein therapeutic strategies are calibrated to each patient’s unique endotype, baseline microbiome configuration, and comorbidity profile. Achieving this ambition requires sustained investment in basic mechanistic research, methodological standardization, and collaborative clinical investigation spanning diverse populations and healthcare settings. The coming decade will determine whether the gut-lung axis fulfills its promise as a transformative target for asthma management or remains an intriguing but clinically elusive biological phenomenon.

## 7 Conclusion

The gut-lung axis represents a pivotal mechanistic framework that extends asthma pathogenesis beyond traditional airway-centered pathways to encompass systemic immune priming by intestinal microbial communities (Masoli et al., 2004; Heijink et al., 2014; Berkinbayeva et al., 2024; Xie et al., 2024). Gut dysbiosis operates as a functionally relevant contributor to allergic airway inflammation through immune cell polarization, microbial metabolite signaling, and barrier dysfunction, with phenotypic heterogeneity closely mirrored by distinct microbial profiles across asthma endotypes. These mechanistic insights establish a compelling rationale for microbiome-targeted interventions, yet the translational pipeline remains constrained by inconsistent efficacy, strain-specific effects, and the absence of standardized protocols validated across diverse populations and disease stages. For the practicing clinician, this evidence landscape demands cautious interpretation: while probiotics, prebiotics, and FMT demonstrate biological plausibility and preliminary efficacy signals, they should currently be regarded as adjunctive strategies rather than replacements for established pharmacotherapy, with patient selection guided by endotype stratification, baseline microbial status, and comorbidity profiles. Advancing microbiome-based precision medicine in asthma will require sustained investment in causal validation through longitudinal cohorts and mechanistic stratification, multi-omics integration to define actionable microbial mediators, and large-scale randomized controlled trials with hard clinical endpoints tailored to specific asthma phenotypes. The coming decade will determine whether the gut-lung axis fulfills its transformative potential for patients with uncontrolled or refractory disease, or remains an intriguing but clinically elusive biological phenomenon.

## Glossary

*A. muciniphila*: *Akkermansia muciniphila*
AhR: aryl hydrocarbon receptor
AKT: protein Kinase B
*B. breve*: *Bifidobacterium breve*
CCL25: C-C motif chemokine ligand 25
CCR9: C-C motif chemokine receptor 9
DC: dendritic cell
*F. prausnitzii*: *Faecalibacterium prausnitzii*
FMT: fecal microbiota transplantation
Foxp3: forkhead box P3
FXR: Farnesoid X receptor
GATA3: GATA binding protein 3
GPBAR1: G protein-coupled bile acid receptor 1
GPCRs: G protein-coupled receptors
GPR41: G protein-coupled receptor 41
GPR43: G protein-coupled receptor 43
HDAC: histone deacetylase
HIF-1α: hypoxia inducible factor-1α
HLA-DQ: human leukocyte antigen
IFN-γ: interferon-γ
IgA: immunoglobulin A
IgE: immunoglobulin E
IL-10: interleukin-10
IL-11: interleukin-11
IL-12: interleukin-12
IL-13: , interleukin-13
IL-17: interleukin-17
IL-25: interleukin-25
IL-33: interleukin-33
IL-4: interleukin-4
IL-5: interleukin-5
IL-6: interleukin-6
ILC: innate lymphoid cell
ILC2: type 2 innate lymphoid cell
ILC3: type 3 innate lymphoid cell
IPA: indolyl-3-propionic acid
*L. reuteri*: *Lactobacillus reuteri*
*L. rhamnosus*: *Lactobacillus rhamnosus*
LPS: lipopolysaccharide
MLCK: myosin light chain kinase
MyD88: myeloid differentiation primary response protein 88
NF-κB: nuclear factor-κB
PI3K: phosphatidylinositol 3-kinase
PMBL: polyvalent mechanical bacterial lysate
PXR: pregnane X receptor
RORγt: retinoic acid receptor-related orphan receptor γt
SCFAs: short-chain fatty acids
STAT6: signal transducer and activator of transcription 6
SUCNR1: succinate receptor 1
TGF-β: transforming growth factor beta
Th1: T helper 1 cell
Th17: T helper 17 cell
Th2: T helper 2 cell
TLR2: Toll-like receptor 2
TLR4: Toll-like receptor 4
TLR5: Toll-like receptor 5
TNF-α: tumor necrosis factor-α
Treg: regulatory T cell
TRPV1: transient receptor potential vanilloid 1
TSLP: thymic stromal lymphopoietin
ZO-1: zonula occludens-1
